# A reassessment of several erstwhile methods for isolating DNA fragments from agarose gels

**DOI:** 10.1007/s13205-021-02691-1

**Published:** 2021-02-23

**Authors:** Xia Gao, Keyin Zhang, Tianzhu Lu, Yan Zhao, Haiyan Zhou, Yanqin Yu, Lucas Zellmer, Yan He, Hai Huang, Dezhong Joshua Liao

**Affiliations:** 1 Department of Pathology, Guizhou Medical University Hospital, 4 Beijing Road, Guiyang, 550004 Guizhou People’s Republic of China; 2 Key Lab of Endemic and Ethnic Diseases of the Ministry of Education of China in Guizhou Medical University, Guiyang, 550004 Guizhou People’s Republic of China; 3 Department of Stomatology, School of Stomatology, Guizhou Medical University, 4 Beijing Road, Guiyang, 550004 Guizhou People’s Republic of China; 4 Center for Clinical Research, Guizhou Medical University Hospital, 4 Beijing Road, Guiyang, 550004 Guizhou People’s Republic of China; 5 Masonic Cancer Center, University of Minnesota, Minneapolis, MN 55455 USA; 6 Center for Clinical Laboratories, Guizhou Medical University Hospital, 4 Beijing Rd, Guiyang, 550004 Guizhou People’s Republic of China

**Keywords:** DNA extraction, Agarose gel, Nested PCR, Reverse transcription

## Abstract

Molecular biology research often requires extraction of DNA fragments from agarose gels. In the past decades, there have been many methods developed for this purpose. Currently most researchers, especially novices, use commercial kits for this extraction, although these kits cost money and the procedures involved are not necessarily easier than some erstwhile methods. We herein reintroduce and reassess several simple and cost-free older methods. One method involves excising a slice of the gel containing the DNA fragment, followed by a thaw-and-freeze procedure to release the DNA from the gel slice into the gel-making buffer. The second method involves a dialysis tubing and requires electroelution of the DNA from the gel slice in the tubing. The third one is to centrifuge the gel slice to release the DNA. The fourth method requires electro-transfer of the DNA from the gel into a filter paper, while the fifth one includes either allowing the DNA in the slice to be dissolved into a buffer or dissolving the DNA-containing gel slice, followed by DNA precipitation with ethanol or isopropanol. The strengths and weaknesses of these methods are discussed to assist researchers in making their choice. We also point out that some of the end uses of the DNA fragment in the agarose gel may not actually require extraction of the DNA. For instance, a tiny DNA-containing gel block or filter paper can be directly used as the template in a nested or semi-nested polymerase chain reaction to preliminarily determine the identity of the DNA fragment.

## Introduction

In molecular biology research, there are many reasons for isolating a DNA fragment from an agarose gel. For instance, molecular biologists often fractionize one or multiple DNA fragments by their molecular mass (weights, often described as “sizes”) in an agarose gel and visualize the fragment(s) as band(s) under ultraviolet (UV) light illumination by staining the DNA with fluorescent dyes like ethidium bromide (Smith 1993; Hamelin and Yelle 1990; Makovets 2013; Upcroft and Upcroft 1993). One or more of the DNA bands may need to be isolated for further analysis or use. For this purpose, a gel slice containing the DNA band of interest will be excised using a razor or surgical blade. The DNA fragment inside the gel slice can then be extracted from the agarose using different methods that have been reported and reviewed in the literature since the 1970s (Moore et al. 2001; Matitashvili and Zavizion 1997; Yu et al. 2009; Pun and Kam 1990; Pollman and Zuccarelli 1989; Hegen 1994; Downey 2003; Finkelstein and Rownd 1978; Girvitz et al. 1980; McDonell et al. 1977; Parker and Seed 1980; Tabak and Flavell 1978; Wheeler et al. 1977; Perlman and Huberman 1977). These methods include the processes of electroelution (Pun and Kam 1990; Pollman and Zuccarelli 1989), glass bead extraction (Pun and Kam 1990), nanoparticle extraction (Saiyed et al. 2007), filter paper extraction (Kunhareang et al. 2010; Grey and Brendel 1992), DNA-binding column extraction (Moore et al. 2001; Yu et al. 2009), affinity chromatographic extraction (McEnery et al. 1986), elution-by-diffusion (Pun and Kam 1990), gel dissolving with a solution (Pun and Kam 1990), etc. Some of these methods share certain steps or reagents. Some methods have been developed as commercial products or have commercial potentials (Moore et al. 2001; Hegen 1994; Downey 2003; Willis et al. 1990; McEnery et al. 1986).

Although there have been many methods reported, today most researchers, especially neophytes, use commercial kits to extract DNA from excised gel slices following manufacturers’ protocols. Most of these kits have evolved from methods reported in the literature and require heating the gel slice to melt the agarose, so that the DNA is released into the buffer used to make the gel. In most cases, the gel-making buffer is either TAE [for it contains Tris(hydroxymethyl)aminomethane (Tris) and Ethylene Diamine Tetraacetic Acid (EDTA)] or TBE (for it contains Tris, Boric acid, and EDTA). After melting the gel, a specific buffer in the kit will be added in, followed by running the DNA-containing solution through a column, a filter, glass beads, etc., to remove the agarose and salts but retain the DNA. Another buffer in the kit is then used to elute the DNA from the column, the filter, the beads, etc. Although the kit suppliers have already optimized the procedures and reagents and provided detailed protocols, the rate of DNA recovery may still vary among different users with different levels of knowledge and experience. Notwithstanding, these commercial kits are easy to use and generally give rise to a high rate of DNA recovery. However, these commercial kits cost money and are not necessarily easier than some earlier methods. Herein we reintroduce and reassess several old-days methods, not only because they are the basic steps or principles of some commercial kits and thus help researchers in fathoming the relevant principles, but also because some of them are still used by us and some others, especially those in developing countries or with limited resources for research.

## Brief outlines of several old-days methods

### Thaw-and-freeze method

Put the DNA-containing gel slice into an Eppendorf tube (preferably a small one) and then heat the tube to melt the agarose gel and release the DNA into the gel-making buffer, usually TAE or TBE as abovementioned. We use a pair of forceps to put the tube into boiling water for a few seconds to quickly melt the gel, but others heat the gel to a temperature varying between 45 and 65 °C for different durations (Kunhareang et al. 2010; Kurien et al. 2001; Pusch 1997; Pun and Kam 1990) or simply use a low-melting agarose (Pun and Kam 1990; Steck 1994; Via and Falkinham III 1991). For regular agarose, the melting temperature and solidifying temperature are 87±1.5 °C and 37±1.5 °C, respectively (whereas the gel made of a low-melting agarose may melt at about 25 °C). After the gel slice has melted and the DNA has been released into the gel-making buffer, immediately put the tube into a freezer to quickly freeze the agarose. Quickly spin down the re-solidified agarose with a mini-centrifuge at room temperature, and then aspirate the DNA-containing supernatant into a new tube. This thaw-and-freeze procedure may be repeated once or twice, which may increase DNA recovery. In most cases, DNA is fractionated in a 1–2% agarose gel, meaning that 98–99% of the gel slice is actually the gel-making buffer, which usually is also the buffer used for electrophoresis. If needed, the DNA-containing buffer can be run through a sephadex size-exclusion column to desalt the buffer. In the old days, the column was often reused several times as it could be washed with a low concentration of HCl after each use. Alternatively, the DNA in the buffer can be extracted by filtrating through a low-binding 0.45-µM cellulose acetate centrifuge filter (Sokolov and Prockop 1994) or using phenol-chloroform to extract the agarose and using ethanol or isopropanol to precipitate the DNA, followed by suspension of the DNA pellet with a Tris-EDTA (TE) buffer (Parker and Seed 1980) or with molecular-grade water. Thawing the gel and using a centrifuge filter or DNA-binding column are actually some of the steps and principles used in many commercial kits.

### Dialysis tubing (or electroelution) method

Put the DNA-containing gel slice into a dialysis tubing with its molecular-weight cutoff smaller than that of the DNA fragment. Fill the tubing with a clean electrophoresis buffer (TAE or TBE) to cover the gel slice and then clip both ends of the tubing. Fix the tubing in the electrophoresis tank so that it cannot move, and then continue electrophoresis for 5–10 min until the DNA has completely migrated from the gel into the buffer, followed by collection of the buffer from the tubing (McDonell et al. 1977). Usually, the volume of the buffer is too large and thus the DNA concentration is too low. Therefore, this method often requires precipitation of the DNA with ethanol or isopropanol and suspension of the DNA pellet in a small volume (20–30 µl) of water or a 1-mM or 10-mM TE buffer (pH 7.4). These additional steps not only concentrate the DNA but also remove the TAE or TBE from the DNA, thus preventing the possible disturbance of the TAE or TBE in the subsequent applications of the DNA, such as sequencing or ligation to a vector.

### Gel-centrifugation method

**Fig. 1:**
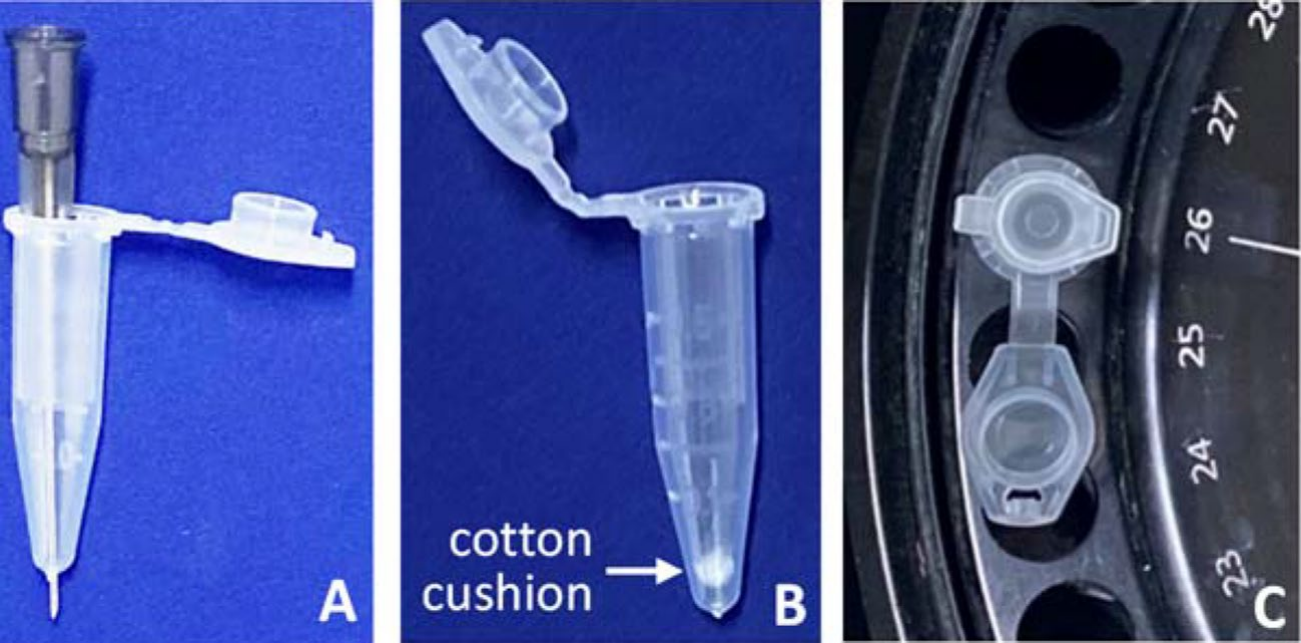
Illustration of the preparation for gel centrifugation. **a** Use a syringe needle to puncture a hole at the bottom of a 500-µl Eppendorf tube. **b** Put a tiny amount of defatted cotton or glass wool at the bottom of the tube as a cushion (the white spot inside the tube), and then lay the DNA-containing gel slice onto it (not shown). **c** Cap the tube and put it into a 1.5-ml Eppendorf tube. Put this small-tube-containing tube into a centrifuge and run it at 5000–10,000 rpm for 5–10 min at room temperature to spin out the DNA-containing gel-making buffer, which will leak into the large tube

The DNA-containing gel slice can be centrifuged to release the DNA, as we have detailed before (Sun et al. 2012): Use a syringe needle to puncture a hole in the bottom of a 500-µl Eppendorf tube, and then lay a tiny amount of glass wool or defatted cotton into the tube as a cushion (Fig. 1), although other researchers make the cushion slightly differently (Wang and Rossman 1994). After laying the DNA-containing gel slice onto the cushion, cap the tube and put it into a 1.5-ml Eppendorf tube without capping it. Put this small-tube-containing tube into a centrifuge and spin it at 5000–10,000 rpm (revolutions per minute) for about 5–10 min at room temperature, although others have used different centrifugation conditions (Barbieri et al. 1996; Wang and Rossman 1994; He et al. 1992). The actual revolution and duration may vary if the size of the gel slice is too large or the gel has 2% or more of agarose. Because a lower temperature makes the gel harder and thus more difficult to release the DNA, it is important to set the centrifuge’s temperature at 25–29 °C or run the centrifuge at room temperature. After centrifugation, the DNA, along with the gel-making buffer, will have been separated from the agarose and leaked into the large tube, while the agarose should be retained in the cushion inside the small tube.

### Filter-paper method

**Fig. 2:**
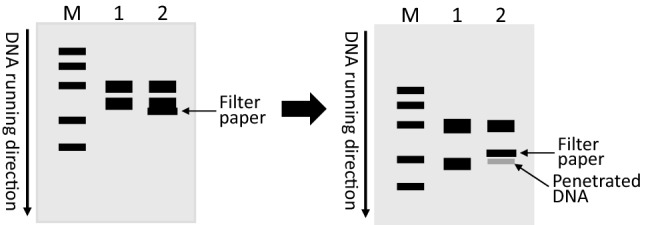
Illustration of the filter-paper method. Suppose that a PCR product is equally divided and loaded into two wells (wells 1 and 2) of an agarose gel, followed by electro-fractionization with the direction of DNA migration shown by the vertical arrow. A molecular weight marker (M), commonly referred to as a “DNA ladder”, is included to indicate the differences in the molecular weights (unspecified here). Suppose that the result of the electrophoresis shows that the PCR yields two amplicons (bands, indicated by thick lines) with one smaller in size than, and thus locating below, the other. If the lower band is the one of interest and is to be isolated, a surgical blade is used to make a cut in the gel immediately below this band in lane 2, followed by insertion of a piece of 3MM Whatman filter paper into the cut (the indicated thin line), with the lane 1 untouched as a control (the left panel). After the electrophoresis continues for a few more minutes, the lower-band DNA in lane 2 has entered into the filter paper, probably with some having penetrated through the paper (the right panel). The same band in lane 1 has migrated further to the same position as, or slightly lower than, the penetrated faint band in lane 2, since the filter paper might slightly retard the DNA migration (the right panel). Take the filter paper out, excise the part containing the DNA, and put it into an Eppendorf tube containing a 1-mM or 10-mM TE buffer (pH 7.4–8.0) for DNA elution by vortexing the tube or spinning it with a mini-centrifuge

During the electro-fractionation of DNA fragment(s) in an agarose gel, when the DNA band of interest has migrated to an ideal position, stop the electrophoresis and use a surgical blade to make an insertion immediately below the DNA band of interest. Insert a sliver of a 3MM Whatman filter paper into the cut (Fig. 2). Some researchers may pre-wet the paper with the electrophoresis buffer before inserting it, but a wet paper is more difficult to insert and a paper actually gets wet instantly inside the gel and the buffer. Continue electrophoresis for a few minutes until the DNA has migrated into the paper, which can be discerned lucidly under UV light. Some of the DNA may penetrate through the filter paper, depending on the filter paper and the electrophoresis conditions (such as the voltage and duration) used; therefore, two layers of the filter paper may be used. Take the paper out, excise the part containing the DNA under a portable torch of UV light and, if needed, shred the DNA-containing part into smaller pieces. Put the paper or paper shreds into an Eppendorf tube containing 20–30 µl of a 1-mM or 10-mM TE buffer (pH 7.4–8.0) or water. Vortex the tube or spin it with a mini centrifuge for tens of seconds to elute the DNA. In this method, a diethylaminoethyl-cellulose (often dubbed as DEAE-cellulose) membrane or another type of cellulose membrane may be used to replace the filter paper (Girvitz et al. 1980). In this case, the DNA cannot run through the membrane during the electrophoresis but will later be more difficult to elute. The DNA on the cellulose membrane can also be used for a Southern blot hybridization with an oligo probe.

### Dissolving gel or DNA method

The DNA-containing gel slice can be crushed or chopped into fine particles in 1 ml of a TE buffer (40 mM Tris and 1 mM EDTA, pH 7.5–8.0). Transfer these gel particles along with the buffer into 5 ml of another buffer containing 40 mM Tris-acetate and 10 mM NaCl, followed by incubation at 37 °C for several hours to allow the DNA to be dissolved into the buffer (Pun and Kam 1990). The eluted DNA needs to be precipitated with isopropanol. Alternatively, the chopped DNA-containing gel can be dissolved in a solution containing 2 mg/ml sodium iodide at 37 °C for several hours (Vogelstein and Gillespie 1979; Chen and Thomas Jr. 1980), followed using glass beads to absorb the DNA overnight. The DNA may then be eluted from the beads with a solution containing 50 mM Tris (pH 9) and 0.2 M NaCl at 60 °C for 30 min.

## Strengths and weaknesses of the older methods

All of the older methods described above are essentially cost-free. However, each of them not only has its strengths but also has its innate weaknesses. The last method is rarely used today, as it takes hours or even overnight and it is inconvenient to precipitate DNA from a large volume of buffer, although it allows the DNA or the gel slice to be dissolved into a buffer without involving centrifugation. The thaw-and-freeze method is a quick and easy procedure but often yields insufficient quantity and quality of DNA, although sometimes iterations of the thaw-and-freeze procedure may increase the yield while subsequent extraction of the DNA with phenol–chloroform increases the quality. The dialysis tubing method is relatively tedious, and often researchers do not have the right tubing available. Its DNA recovery rate is also relatively low because some DNA may stick to the tubing. These shortcomings may be among the reasons for its less-frequent use by today’s researchers, compared with some other methods described herein.

The gel-centrifugation method shows the highest yield in our lab and is our first choice when a large quantity of high-quality DNA is needed. Its variants described by Wang and Rossman (1994), He et al. (1992), Grey and Brendel (1992), and Pusch (1997) may also produce a high yield of high-quality DNA. However, it may require some practice in making the cotton cushion. According to the feedback we received from some users of this method, it is easy to make a mistake using too much cotton when making the cushion. An unnecessarily larger amount of cotton will retain more DNA (which can be discerned under UV light). Probably, it may be better to use glass wool if it is available, as DNA may not bind to it as tightly as to cotton. In addition, one may inadvertently centrifuge the gel slice at 4 °C preset by a previous user of the centrifuge.

The DNA samples recovered from the first three methods described above are in a gel-making buffer, either TAE or TBE in most cases. Although there are different recipes for making TAE, it is often made as a concentrated solution of 50 times (50x), containing 242 g of Tris and 18.612 g of EDTA or 37.2 g of Na2EDTA.2H_2_O, in addition of 57.1 ml of acetic acid. Usually, 1 × or 0.5 × TAE is used for electrophoresis of DNA; therefore, the working solution (1x) of TAE is actually a relatively high concentration of TE buffer, containing about 40 mM of Tris and 1.3 mM of EDTA. When adding 1–2 µl of the DNA-containing TAE buffer into a volume of 20–25 µl of a polymerase chain reaction (PCR) system, the buffer may not significantly affect the PCR efficacy, according to our experience. However, a too-large amount of the buffer may be adverse for certain subsequent protocols, not just PCR but also DNA sequencing, DNA ligation, etc., mainly because high concentrations of Tris and EDTA are inhibitory to many enzymes. For these latter applications, the DNA can be further purified using ethanol or isopropanol precipitation and then suspension of the DNA pellet in a small (20–30 µl) volume of water or 1-mM or 10-mM TE buffer (pH 7.4–8.0). We sometimes use 0.5 × TAE, but not 1 × TAE, for DNA electrophoresis to avoid the necessity of the subsequent DNA precipitation. Moreover, we do not find any obvious difference between fresh TAE and TBE used for the electrophoresis pertaining to the quality and quantity of the isolated DNA. However, we have a perception, although sans tenable experimental evidence, that the DNA in TAE has less interference with the efficacy of the latter applications when compared with the DNA in TBE. Presumably, the boric acid in TBE may have an additional interference, besides the Tris and EDTA, with the activity of the enzymes used in the subsequent protocols. Since little is known about the effect of boric acid on DNA and on the enzymes commonly used in molecular biology, it seems safer to avoid using TBE.

The filter-paper method is the easiest one to use among the methods we described herein. In our lab, its yield is sufficient for DNA sequencing, ligation to a vector, etc. If the yield from one filter paper inserted into one lane of the gel is still insufficient, the yield can easily be raised using more filter papers inserted into more lanes of the gel, especially when the DNA fragment is a product of a PCR that can be performed in duplicates or triplicates. Another advantage of this tactic is that it only requires making an incision in the gel, whereas the other methods require careful excision of a gel slice and thus expose the researcher to UV light for a much longer time. When we have many DNA bands in a gel to excise, we correct this weakness by taking a photo of the gel under UV light and then using the photo as a ruler to guide the gel excision under normal light, as described by Zimmermann et al. (1998). The best merit of the filter paper method is that the DNA is directly recovered and concentrated in a small volume of TE buffer or water, but not in a large volume of TAE or TBE, and thus can be directly used in any subsequent protocols. A 1-mM or 10-mM TE buffer with a pH of 7.4–8.0 seems better than water for the final elution of the DNA from the filter paper, according to our empirical knowledge, likely because DNA dissolves better in an alkaline solution (but a solution with a too-high pH can destroy DNA).

## Some technical details needed to be considered

Whether a modern method with a commercial kit or one of the several aforementioned older methods is used, some technical details should be considered. One is that it is necessary to remove as much as possible the excess gel, i.e., the part without DNA, from the gel slice before going into the latter steps. The superfluous part of the gel will hamper the DNA release and increase the volume of the resulting DNA-containing buffer, in turn lowering the DNA concentration. Another consideration is that, if possible, a lower percentage of agarose gel should try to be used for fractionization of the DNA fragment(s) in question, which may not be optimal for visualizing the DNA as a sharp band in the gel but is better for isolation of the DNA. Although Smith mentioned that a gel could be made at 0.3% of agarose (Smith 1993), we find that it is relatively easier to make it at 0.5–3% and that DNA in a gel within this range of concentrations can be extracted efficiently using the gel-centrifugation method or the filter-paper one. Unfortunately, a 1–2% agarose gel is most commonly made for visualizing a DNA fragment as a sharp band and, therefore, in realty most DNA-containing gel slices have 1–2% agarose. Nevertheless, if an electrophoresis is performed specifically for DNA extraction, a gel of lower-than-usual percentage should be considered. To facilitate the gel melting and DNA release without making the band image less sharp, we sometimes use a gel made of a mixture of regular agarose and low-melting agarose, with a ratio varying around 1:1, and run it at 4 ˚C.

## Applications with no need to extract DNA from agarose gels

**Fig. 3:**
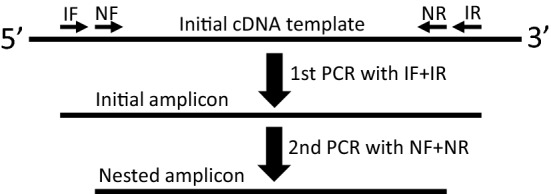
Illustration of the primers for nested or semi-nested PCR. The initial PCR using initial forward (IF) and reverse (IR) primers will produce a DNA amplicon. If this initial amplicon has an unexpected size, it should be further analyzed using a nested PCR with its forward (NF) and reverse (NR) primers located within the amplicon (thus usually dubbed as “inner primers”), which should yield an amplicon (nested amplicon) shorter than the initial one, if the initial one originates from an alternative splicing. Alternatively, the IF may be paired with the NR or the NF may be paired with the IR to run a semi-nested PCR; each of the two semi-nested PCRs may engender a semi-nested amplicon with a size smaller than the initial one but larger than the nested one (not shown)

So often the purpose of extracting a DNA fragment from an agarose gel is to preliminarily authenticate or determine its identity, especially when its size is either smaller or larger than what is anticipated. However, we find that it is not necessary to extract the DNA from agarose gel for this purpose. For instance, when PCR with a complementary DNA (cDNA) produced from reverse transcription (RT), which reflects an RNA, as the template results in an amplicon with a smaller or larger molecular weight, either the amplicon is a nonspecific one derived from another gene or it originates from an unknown RNA variant due to an unknown splicing alternative. To differentiate one from the other, a nested or semi-nested PCR seems to be the most feasible approach, as it offers results in only several hours. Actually, alternative splicing occurs so frequently and probably many alternatives occurring in various cell types and various physiological and pathological situations may not have been known. Therefore, we often initially design four primers when studying RNA expression with RT-PCR approaches, using the tack illustrated in Fig. 3, so that we have primers available for nested and semi-nested PCRs if the size of an amplicon surprises us. This four-primer set can make three nested and semi-nested PCR sets besides the set for the initial PCR (Fig. 3), together proffering us a powerful tack for preliminary determination of the identity of the initial amplicon. These nested and semi-nested PCRs do not require purified DNA from a gel slice as the template. One can simply cut a small block (1 mm^3^ or smaller) of the DNA-containing gel slice (use a portable UV-light torch to ensure that the tiny gel block contains the DNA), and directly put it into the tube of PCR system as the template. During the first step of PCR, which is 4–5 min of incubation at 94–95 ˚C, the gel will melt and release the DNA as the template. However, since regular agarose may inhibit PCR, the gel block should be very small, the PCR volume may be slightly larger (25–50 µl) than usual (our routine PCR volume is 20 µl), or a low-melting agarose should be used. If the initial amplicon (the DNA fragment) is derived from the target gene, the nested PCR or one of the two semi-nested PCRs will likely result in an amplicon with a size smaller than the initial one. However, it remains possible that the region(s) harboring one or both nested primers (NF and NR in Fig. 3) are unfortunately lacked by the RNA variant; in this case, the nested PCR and one or both of the two semi-nested PCRs will fail to produce the anticipated amplicon. If the initial amplicon is nonspecific, none of the nested and semi-nested PCRs will yield an amplicon either.

An alternative involves the filter paper method. Once the DNA-containing filter paper is taken out from the gel, cut a small piece of the paper and directly put it into the tube of PCR system as the template, i.e., replace the abovementioned DNA-containing tiny gel block with a sliver of the DNA-containing paper. During the PCR, some DNA will be released from the paper as the template (Kunhareang et al. 2010). This alternative method seems better, in our experience, presumably because regular agarose has certain inhibitory effects on PCR.

## Conclusions

We reintroduce and reassess several erstwhile methods of DNA extraction from agarose gels. In our opinion, these methods are cost-free, and some of them are equally simple, if not simpler, when compared with the procedures involved in some commercial kits. However, each of these older methods not just has its merits but also has its shortcomings. Researchers should balance the advantages against the disadvantages of each method and compare one with another, including one of the commercial kits, when deciding whether to use a commercial kit or a method outlined herein. Researchers should also bear in mind that there are certain end uses of the DNA fragment inside the agarose gel that do not really require extraction of the DNA, such as direct use of a tiny DNA-containing gel block or filter paper as the template to perform a nested or semi-nested PCR to preliminarily determine the identity of the DNA fragment.

## Data Availability

Not applicable as this is a review article without presenting any data.
